# Gr-1^+^CD11b^+^ Immature Myeloid Cells (IMC) Promote Resistance of Pro-Inflammatory T Cells to Suppression by Regulatory T Cells in Atherosclerotic Apo E- Deficient Mice

**DOI:** 10.1371/journal.pone.0108620

**Published:** 2014-09-30

**Authors:** Yulin Chen, Ying Jian, Minjie Liu, Liang Zhong, Fang Zhang, Weifeng Yang, Zhao Xu, Guofan Chen, Yuhua Liu

**Affiliations:** 1 Department of Cardiology, The Affiliated Hospital of Hangzhou Normal University, Hangzhou, China; 2 Department of Clinical Laboratory, The Affiliated Hospital of Hangzhou Normal University, Hangzhou, China; Centre de Recherche Public de la Santé (CRP-Santé), Luxembourg

## Abstract

Accumulating evidence indicates that both defects in Treg numbers and/or function as well as resistance of effector T cells to suppression may contribute to the development of human chronic inflammatory diseases. However, which mechanism involved in the progression of atherosclerosis remains unclear. In this study, we evaluated the production and function of CD4^+^ inflammatory and regulatory T cells in atherosclerosis-prone mice. We found that the hyperactivity and unresponsiveness to Treg-mediated suppression of inflammatory CD4^+^ T cells occurred in the progression of atherosclerosis, though Treg cells were present in very large numbers and fully functional. We further found that Gr-1^+^CD11b^+^ immature myeloid cells were significantly accumulated in atherosclerotic Apo E^−/−^ mice, and they promoted resistance of inflammatory CD4^+^ T cells to Treg-mediated suppression in vitro and in vivo. we further confirmed that Gr-1^+^CD11b^+^ immature myeloid cells produced high level of interleukin 6 which was at least partially responsible for inducing unresponsiveness of inflammatory CD4^+^ T cells to suppression via activation of Jak/Stat signaling pathway. Taken together, these findings might provide new insights to explore potential targets for immune therapeutic intervention in atherosclerosis.

## Introduction

Atherosclerosis is the common pathological process underlying coronary arterial disease (CAD), carotid stenosis, and peripheral arterial disease, which is one of the major cause of death and disability worldwide [1]. Accumulating evidence suggests vascular wall chronic inflammation mediated by CD4^+^ T cells plays a critical role in the development and progression of atherosclerosis [2]. Several studies indicated that Th1 cells had a proatherogenic role since blocking Th1 polarization by pentoxifylline significantly attenuated atherosclerotic lesion development in experimental atherosclerosis mice model [3]. In addition, it has been reported that Th17 cells were also deeply involved in the development of atherosclerotic lesions [4], [5]. The up-regulation of Th17 response was observed in both local atherosclerotic plaque and circulating lymphocytes which accelerated atherosclerotic lesion formation. Furthermore, IL-17A antibody treatment markedly reduced both area and vulnerability of the atherosclerotic plaque in atherosclerosis prone models [6]. Furthermore, regulatory T cells (Treg), as one of the main cell populations responsible for maintaining immune homeostasis, play a crucial role in the regulation of pro-inflammatory T cell responses and have the protective effects on the development of atherosclerosis [7], [8]. A fine balance between effector T cells and Treg cells is thought to be crucial in regulating immune homeostasis and the prevention of inflammatory and autoimmune diseases [9]. Considerable evidence supports that high levels of pro-inflammatory cytokines lead to the occurrence of effector/regulatory T-cell imbalance in chronic inflammatory diseases. Nevertheless, the underlying mechanism remains unclear.

Gr-1^+^CD11b^+^ immature myeloid cells (IMC) represent a heterogeneous population of myeloid cells in early differential stages that comprises immature macrophages, granulocytes and dendritic cells [10]. Currently, most of observations on the role of these cells in regulating immune responses come from studies in the field of cancer research. They are also called myeloid-derived suppressor cells (MDSC) because that it has consistently been shown that these cells have a remarkable ability to suppress T-cell response through producing Arginase 1 (ARG1), inducible nitric oxide synthase (iNOS) and Transforming growth factor beta 1 (TGF-β1) in tumor-bearing mice [11], [12], [13]. Cells with a similar phenotype were also observed in several inflammatory and autoimmune diseases [14], [15], however, there is an ongoing debate about the role of these cells in chronic inflammatory diseases. In alopecia areata, a mild autoimmune disease that affects hair follicles, IMC had the potential to suppress auto-reactive T cell proliferation. but in the SLE mouse model, these cells had the ability to immune stimulatory. It still remains to be elucidated whether and how IMC are involved in the pathogenesis of atherosclerosis.

Apolipoprotein E (Apo E) deficient mice is a particularly popular model for investigating the immunologic mechanisms involved in the pathogenesis of atherosclerosis because it spontaneously develop atherosclerotic lesions in the aorta that similar to human atherosclerosis even on a standard chow diet [16]. In the current study, we found that the frequencies of both Th1 and Th17 cells in the spleen of Apo E^−/−^ mice increased in parallel to the rise in the serum level of total cholesterol and interleukin 6 (IL-6). Unexpectedly, Treg cells were also present in large numbers in atherosclerotic Apo E^−/−^ mice and the immunosuppressive capacity of Treg cells isolated from atherosclerotic Apo E^−/−^ mice was comparable to the counterparts from their age matched wild-type littermates. However, the proliferation and cytokine production of CD4^+^CD25^−^ T cells from atherosclerotic Apo E^−/−^ mice was rarely affected in presence of Treg cells. These data indicated that the hyperactivity of inflammatory CD4^+^ T cells in atherosclerotic Apo E^−/−^ mice were due to unresponsiveness to Treg-mediated suppression. We further found that IMC were significantly accumulated in atherosclerotic Apo E^−/−^ mice, and they promoted resistance of inflammatory CD4^+^ T cells to Treg-mediated suppression in vitro and in vivo. We further confirmed that IMC produced high level of IL-6 which was at least partially responsible for inducing unresponsiveness of inflammatory CD4^+^ T cells to suppression via activation of Jak/Stat signaling pathway because both depletion of IMC or blockade of Jak/Stat signaling pathway significantly suppressed the activation of pro-inflammatory T cells in vivo. Taken together, these findings might provide new insights to explore potential targets for immune therapeutic intervention in atherosclerosis.

## Materials and Methods

### Animals, cell lines and reagents

Apo E^−/−^ mice on a C57BL/6 background and their wild type littermates (4-week-old) were purchased from Peking University Laboratory Animal Center and maintained under a specific pathogen-free condition. Experiments and animal care were performed according to protocols approved by Hangzhou Normal University Institutional Animal Care and Use Committee. EL-4 T lymphoma was obtained from ATCC. INF-γ, IL-6, IL-17A and TGF-β1 ELISA kits were purchased from eBioscience. Cytofix/Cytoperm kit was obtained from BD Bioscience. CP690550 was purchased from Invivogen. [methyl-3h] thymidine was from GE Healthcare Life Sciences.

### Flow cytometry

Flow cytometry was conducted using a BD Biosciences FACSCalibur device and analyzed with FlowJo (Tree Star). Abs used for FACS staining were anti-mouse CD4, IL-17A, IFN-γ, FoxP3, Gr-1, and CD11b from eBioscience. To detect FoxP3 and intracellular cytokine, Before staining, cells were fixed and permeabilized by Cytofix/Cytoperm kit according to the manufacturer’s instructions.

### Cell isolation

A single-cell suspension was prepared from the spleen and red cells were removed using ACK lysing buffer. Erythrocyte-depleted lymphocytes were staining with anti-mouse CD4 FITC and CD25 PE for Treg, Gr-1 FITC and CD11b PE for IMC, then isolated by cell sorting on a FACSAria II cell sorter (BD Biosciences). The purity of the cell populations were more than 99%.

### ELISA and total serum cholesterol analysis

INF-γ, IL-6, IL-17A and TGF-β1 levels were measured according to the manufacturer’s instructions. Total serum cholesterol was analyzed with a standard method by auto-clinical chemistry analyzer (Hitachi 917).

### Suppression assay

CD4^+^CD25^−^ T cells from 20-week old C57BL/6 mice or Apo E^−/−^ mice were used as effector cells and cultured at 60000 cells per well in 200 µL culture volume that stimulated with 3 µg/ml CD3 and 1 µg/ml CD28, in the presence or not of indicated numbers of Treg cells for 3 days. 1 µCi of [3H] thymidine was added in each well 18 hrs before harvest, T cell proliferation was determined by [3H] thymidine incorporation according to the manufacturer’s instructions. For in vivo suppression assay, atherosclerotic Apo E^−/−^ mice were pre-treated i.p. with 100 µg RB6-8C5 mAb or PBS twice a week before adoptive transfer of 1×10^7^ CFSE-labeled CD4^+^CD25**^−^** T cells. Three days after transfer, mice were sacrificed and CFSE dilution was analysis by flow cytometry.

### RNA isolation and real-time quantitative PCR

Total RNA was isolated using TRIzol reagent (Invitrogen) according to the manufacturer’s instructions. Real-time quantitative RT-PCR analysis was performed using an ABI 7500 system (Applied Biosystems). The relative expression level of each gene was normalized to GAPDH.

### Western blot

Total cell lysates were subjected to SDS-PAGE and transferred to PVDF membranes, then immunoblotted with antibodies against total and phosphorylated Stat1, Stat3, Erk, respectively, followed by hybridization with the secondary HRP-conjugated Abs. The housekeeping gene (β-action) acts as a control. All Abs were obtained from Cell Signaling Technology.

### Statistical analysis

Statistical analysis was performed using Student’s t test and analysis of variance (anova) using SPSS (v 19.0). P value <0.05 considered statistically significant.

## Results

### 1. Systemic immune dysregulation occurs in atherosclerotic Apo E^−/−^ mice

**Figure 1:**
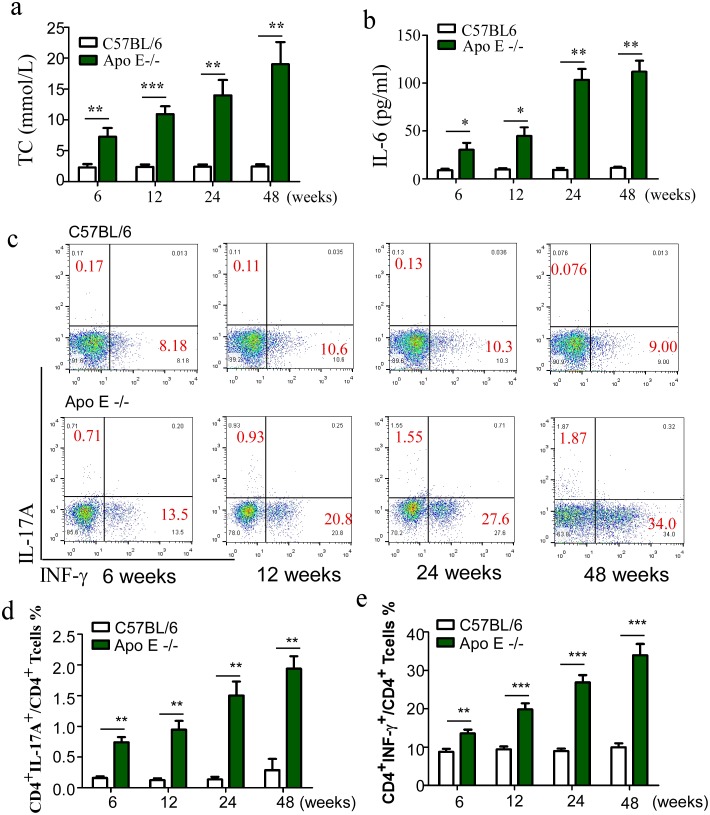
The frequencies of both Th1 and Th17 cells in the spleen of Apo E^−/−^ mice increase in parallel to the rise in the serum level of total cholesterol and IL-6. (a), the serum level of Total cholesterol levels in Apo E^−/−^ mice were measured compared with age-matched C57BL/6 mice. Data are the mean ± SEM (n = 4) of one representative experiment. **p<0.01, ***p<0.001. (b), the serum level of IL-6 in Apo E^−/−^ mice and age-matched C57BL/6 mice was measured by ELISA. Shown is one representative experiment of three performed. *p<0.05, **p<0.01. Apo E^−/−^ mice were fed with standard chow diet and sacrificed at age 6, 12, 24, and 48 weeks. Splenocytes were stained with FITC-CD4, PE-IL-17A and APC-IFN-γ antibody, (c), Representative plots are shown. The frequencies of Th1and Th17 cells are shown in (d) and (e), respectively. Data are the mean ± SEM (n = 4) of one representative experiment out of three performed. **p<0.01, ***p<0.001.

**Figure 2:**
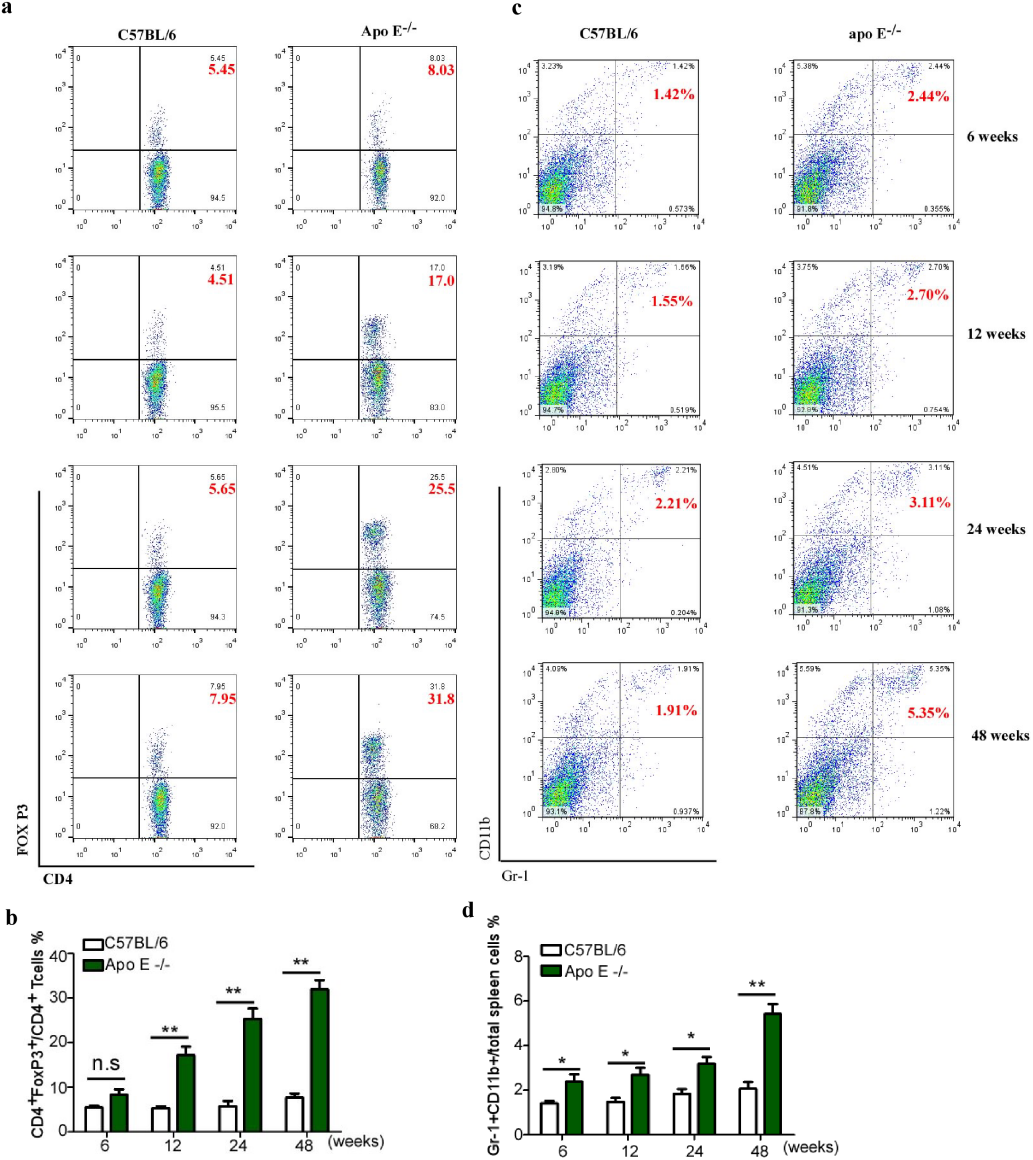
The accumulation of Treg and IMC in the spleen of Apo E^−/−^ mice. (a), Splenocytes were stained with FITC-CD4 and PE-FoxP3 antibody, the frequencies of Treg cells at time points mentioned above were measured by flow cytometry. Representative plots are shown in (b). Data are the mean ± SEM (n = 4) of one representative experiment out of three performed. (c), Splenocytes were stained with FITC-Gr-1 and PE-CD11b antibody, the frequencies of IMC in the spleen at indicated time points were measured by flow cytometry. Representative plots are shown in (d). Data are the mean ± SEM (n = 4) of one representative experiment out of three performed. n.s = not significant, *p<0.05, **p<0.01.

To investigate that the immunologic mechanisms involved in the pathogenesis of atherosclerosis, Apo E^−/−^ mice, a well-known spontaneous hypercholesterolemia and atherosclerosis model, were fed with a regular diet over a 48-week period, the serum level of total cholesterol and IL-6 as well as the proportion of two atherosclerosis related pro-inflammatory (Th1 and Th17 cells) and regulatory T cell subsets were examined at age 6, 12, 24 and 48 weeks. Consistent with previous reports [9], a continuous increase in the serum level of total cholesterol was observed in Apo E^−/−^ mice over the 48-week period (Fig. 1a). In addition, IL-6, a well-known pro-inflammatory cytokine involved in the pathogenesis of atherosclerosis, was dramatically up-regulated, whereas the serum total cholesterol and IL-6 levels kept stable over the 48-week period in wild type littermates (Fig. 1b). We further found that the proportion of two atherosclerosis related pro-inflammatory T cell subsets in spleen continued to rise from week 6 to week 48 in Apo E^−/−^ mice (Th1: from 13.59±0.96 to 33.95±2.93; Th17: from 0.74±0.08 to 1.94±0.20) but no significant increase over the 48-week period in wild type littermates (Fig. 1c, 1d, and 1e). To investigate whether the activation of these atherosclerosis related pro-inflammatory T cells was due to the decreased numbers of immune suppressive cells, we detected the levels of Treg and IMC in the spleen of Apo E^−/−^ mice. As shown in Fig. 2, along with the increased level of atherosclerosis-related effector cells, Treg and IMC unexpectedly accumulated in the spleen of Apo E^−/−^ mice that appeared to closely relate to the progression of atherosclerosis (Treg: from 6.58±0.77 to 30.00±5.53; IMC: from 2.38±0.33 to 5.42±0.45).

### 2. Resistance of pro-inflammatory T cells to suppression, instead of impaired Treg cells, contributes to the ongoing inflammatory response in atherosclerotic Apo E^−/−^ mice

**Figure 3:**
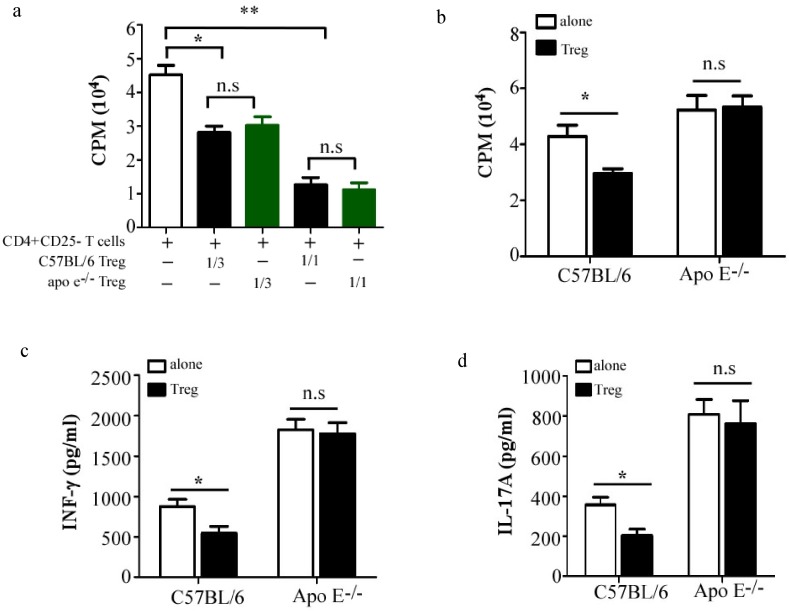
Resistance of pro-inflammatory T cells to suppression, instead of impaired Treg cells, contributes to the ongoing inflammatory response in atherosclerotic Apo E^−/−^ mice. (a), 6×10^4^ CD4^+^CD25^−^ T cells from C57BL/6 mice were stimulated with 3 µg/ml CD3 and 1 µg/ml CD28, in the presence or not of indicated numbers of Treg from 20-week old Apo E^−/−^ mice or age-matched C57BL/6 mice for 3 days. 1 µCi of [3H] thymidine was added in each well 18 hrs before harvest, T cell proliferation was determined by [3H] thymidine incorporation. Data are the mean ± SEM (n = 4) of one representative experiment out of three performed. CD4^+^CD25^−^ T cells isolated from 20-week old Apo E^−/−^ mice or age-matched C57BL/6 mice were cultured alone or with Treg cells isolated from 20-week old Apo E^−/−^ mice at 3∶1 ratio, T cell proliferation was examined as shown in (b). The amounts of IFN-γ (c) and IL-17A (d) in supernatant were measured by ELISA (n = 4). Data are representative of three independent experiments. n.s = not significant; *p<0.05, **p<0.01, ***p<0.001.

To determine whether the suppressive activity of Treg was impaired, thereby resulting in uncontrollable differentiation and proliferation of Th1 and Th17 in atherosclerotic Apo E^−/−^ mice, CD4^+^CD25^−^ T cells from C57BL/6 mice were cultured with or without Treg cells isolated from either 20-week old atherosclerotic Apo E^−/−^ or age-matched C57BL/6 mice at indicated ratio in the presence of anti-CD3 and anti-CD28 antibodies. As shown in Fig. 3a, both Treg cells significantly inhibited the proliferation of CD4^+^CD25^−^ T cells from C57BL/6 mice, but no significant difference could be found between Treg from atherosclerotic Apo E^−/−^ and C57BL/6 mice, which indicated the intrinsic suppressive ability of Treg cells from atherosclerotic Apo E^−/−^ mice remained intact. To investigate whether resistance of pro-inflammatory T cells to Treg-mediated suppression exists in atherosclerotic Apo E^−/−^ mice, CD4^+^CD25^−^ T cells from atherosclerotic Apo E^−/−^ mice or age-matched C57BL/6 mice were co-cultured with Treg isolated from 20-week old atherosclerotic Apo E^−/−^ mice for 3 days. We found that both proliferation (Fig. 3b) and cytokine production (Fig. 3c, 3d) of CD4^+^CD25^−^ T cells from C57BL/6 mice was significantly inhibited when co-cultured with Treg, however, the activation of CD4^+^CD25^−^ T cells from atherosclerotic Apo E^−/−^ mice was rarely affected in presence of Treg cells. These data strongly indicated that the ongoing inflammatory response in atherosclerotic Apo E^−/−^ mice was attributable to resistance of pro-inflammatory T cells to suppression.

### 3. IMC mediate unresponsiveness of pro-inflammatory T cells to suppression

**Figure 4:**
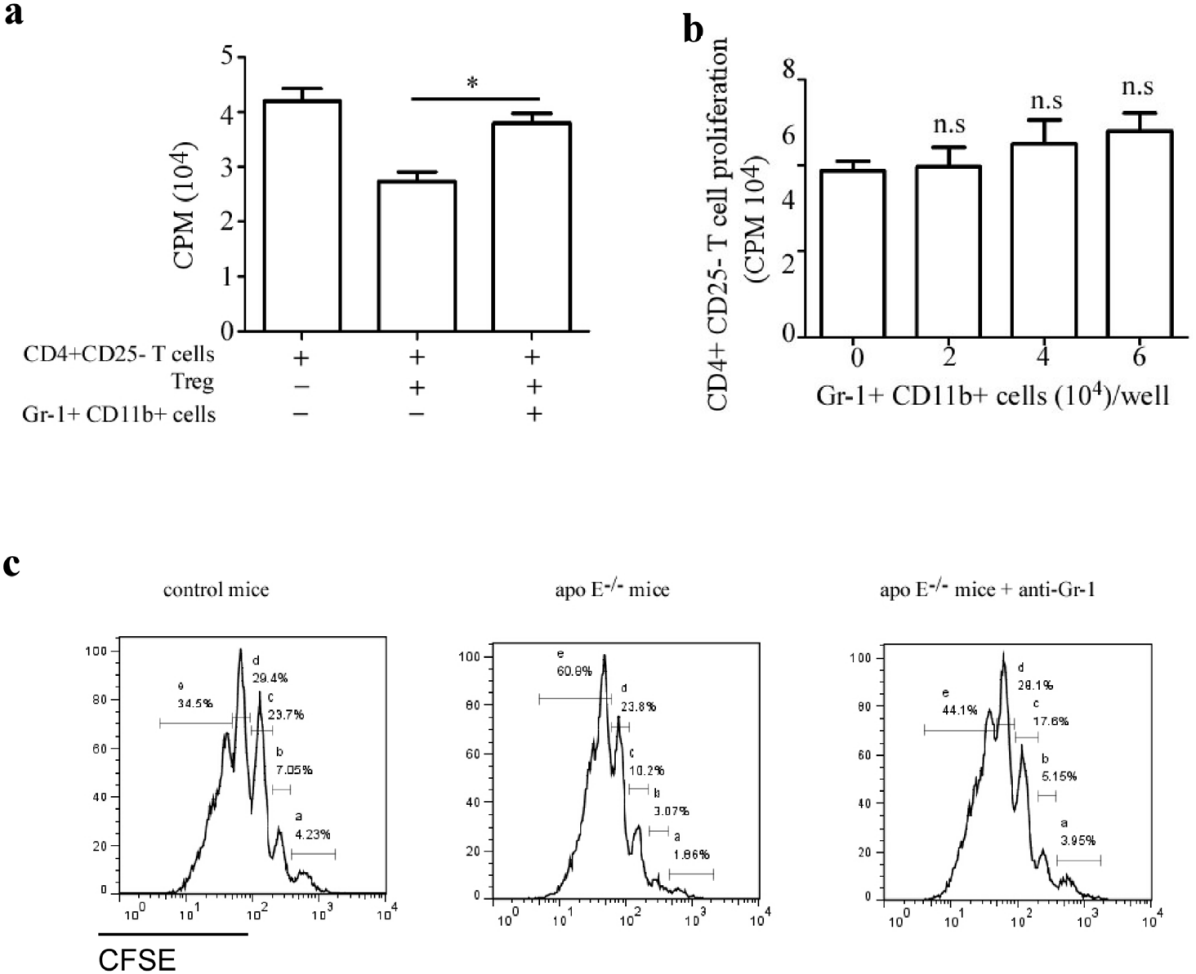
Accumulated Gr-1^+^CD11b^+^ cells cause unresponsiveness of pro-inflammatory T cells to suppression by Treg cells in Apo E^−/−^ mice. (a), CD4^+^CD25^−^ T cells from C57BL/6 mice were cultured with or without Gr-1^+^CD11b^+^ cells and Treg cells, T cell proliferation was determined by [3H] thymidine incorporation. Similar results were obtained in three independent experiments. (b), 6×10^4^ CD4^+^CD25^−^ T cells from C57BL/6 mice were cultured in the presence of indicated numbers of Gr-1^+^CD11b^+^ cells from 20-week old Apo E^−/−^ mice for 3 days. 1 µCi of [3H] thymidine was added in each well 18 hrs before harvest, T cell proliferation was determined by [3H] thymidine incorporation. Data are the mean ± SEM (n = 4) of one representative experiment out of three performed. (c), atherosclerotic Apo E^−/−^ mice were pre-treated i.p. with 100 µg RB6-8C5 mAb or PBS twice a week before adoptive transfer of 1×107 CFSE-labeled C57BL/6 CD4^+^CD25^−^ T cells. Three days after transfer, mice were sacrificed and CFSE dilution was analysis by flow cytometry. Data are the mean ± SEM (n = 5) of one representative experiment. Similar results were obtained in at least three independent experiments. n.s = not significant; *p<0.05, **p<0.01.

Given IMC were identified as one of the main cell populations responsible for regulating T-cell response in pathological conditions, such as cancer, infectious and inflammatory diseases, we investigated whether the accumulated IMC was involved in regulating unresponsiveness of pro-inflammatory T cells to suppression by Treg. CD4^+^CD25^−^ T cells from C57BL/6 mice were co-cultured with or without Treg and IMC isolated from 20-week old atherosclerotic Apo E^−/−^ mice at 3∶1∶1 ratio for 3 days. The proliferation level of CD4^+^CD25^−^ T cells was significantly inhibited by Treg, however, adding IMC significantly abrogated the Treg suppressive effect on CD4^+^CD25^−^ T cells (Fig. 4a). We further investigated whether IMC were able to directly stimulate T cells, IMC isolated from 20-week old atherosclerotic Apo E^−/−^ mice were co-cultured with CD4^+^CD25^−^ T cells from C57BL/6 mice in the presence of anti-CD3 and anti-CD28 antibodies. As shown in Fig. 4b, adding IMC did not change the proliferation level of CD4^+^CD25^−^ T cells at all indicated ratio. To further confirm the role of IMC in the resistance of pro-inflammatory T cells to suppression in vivo, atherosclerotic Apo E^−/−^ mice were pre-treated i.p. with 100 µg RB6-8C5 mAb to depletion of IMC or PBS twice a week before adoptive transfer of CFSE-labeled C57BL/6 CD4^+^CD25^−^ T cells. Three days after transfer, mice were sacrificed and CFSE dilution was analysis by flow cytometry. As shown in Fig. 4c, more vigorous proliferation of CFSE-labeled T cells were observed in atherosclerotic Apo E^−/−^ mice compared with in C57BL/6 mice, nevertheless, the proliferation level of CFSE-labeled T cells was significantly suppressed in RB6-8C5 mAb-treated Apo E^−/−^ mice. Taken together, these results indicated that the accumulated IMC at least partially caused resistance of pro-inflammatory T cells to suppression by Treg that contribute to the progression of atherosclerosis.

### 4. IMC-induced unresponsiveness of pro-inflammatory T cells to suppression is IL-6 dependent

**Figure 5:**
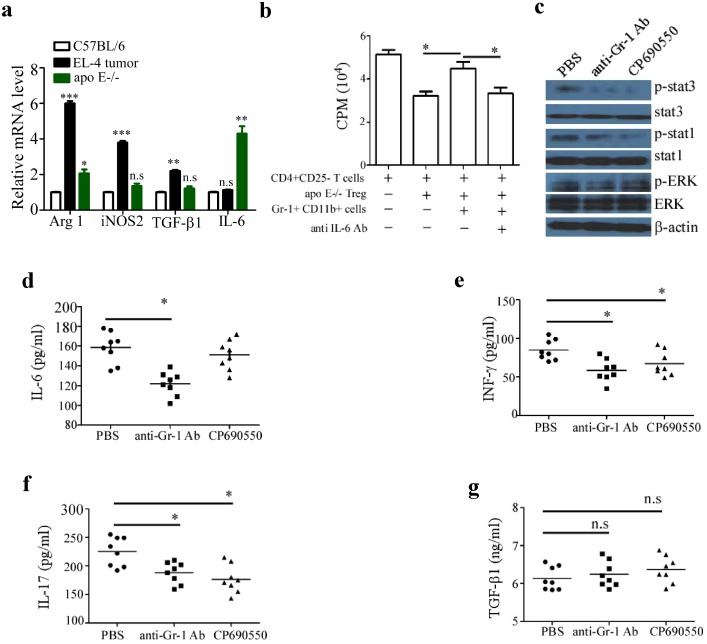
Gr-1^+^CD11b^+^ cell-induced unresponsiveness of pro-inflammatory T cells to suppression is IL-6 dependent. (a), Gr-1^+^CD11b^+^ cells were isolated from the spleen of 20-week old Apo E^−/−^ and C57BL/6 mice and EL-4 tumor-bearing mice. The mRNA expression of Arg 1, iNOS 2, TGF-β1 and IL-6 was measured by qRT-PCR. (b), CD4^+^CD25^−^ T cells from C57BL/6 mice were cultured with or without Gr-1^+^CD11b^+^ cells and Treg cells as well as 20 µg/ml IL-6 antibody, T cell proliferation was determined by [3H] thymidine incorporation. Data are the mean ± SEM (n = 4) of one representative experiment. Atherosclerotic Apo E^−/−^ mice were pre-treated i.p. with 100 µg RB6-8C5 mAb or Jak inhibitor tofacitinib (CP-690,550) 30 mg/kg twice a week, (c), Phosphorylation of stat1 and stat3 as well as Erk was detected by immunoblot. Shown is one representative experiment out of three. the serum levels of IL-6 (d), INF-γ (e), IL-17A (f) and TGF-β1 (g) were measured by ELISA. Data are the mean ± SEM (n = 4) of one representative experiment. Similar results were obtained in at least three independent experiments. n.s = not significant; *p<0.05, **p<0.01.

To investigate which molecular mechanism was involved in IMC mediated unresponsiveness of pro-inflammatory T cells to suppression, we analyzed the RNA expression of four IMC-related immune regulatory molecules. Compared with the MDSC isolated from EL-4 tumor bearing mice, the expression of arginase-1, iNOS2 and TGF-β1 was significantly low but IL-6 level was dramatically high in IMC from atherosclerotic Apo E^−/−^ mice (Fig. 5a). To investigate whether IL-6 was involved in IMC-mediated unresponsiveness of pro-inflammatory T cells to suppression, IL-6 blocking mAb was added into culture medium at 20 µg/ml. As shown in Fig. 5b, blockade of IL-6 markedly restored the responsiveness of pro-inflammatory T cells to Treg mediated suppression in presence of IMC. To determine further whether depletion of IMC or blockade of Jak/Stat signaling pathway could suppress the activation of pro-inflammatory T cells in vivo, atherosclerotic Apo E^−/−^ mice were treated by RB6-8C5 mAb or Jak inhibitor (CP690550) twice a week before they were sacrificed for measuring the phosphorylation level of IL-6 signaling-related transcription factors in CD4^+^ T cells and T cell-related cytokines in serum. As expected, phosphorylation of stat3 and stat1 was completely abolished by CP690550 though the activity of Erk was barely affected. With regard to RB6-8C5 mAb treatment, a similar picture emerged (Fig. 5c). The serum IL-6 level was significantly down-regulated by depletion of IMC (Fig. 5d). Both treatment significantly down-regulated INF-γ and IL-17A level in serum (Fig. 5e, 5f), and up-regulated TGF-β1 level though it didn’t reach statistical significance (Fig. 5 g). Taken together, these results indicated that accumulation of IMC result in high level of circulating IL-6 that mediated unresponsiveness of pro-inflammatory T cells to suppression by Treg that contribute to the atherogenic processes in Apo E^−/−^ mice.

## Discussion

Atherosclerosis is believed to belong to autoimmune diseases, based principally on the evidence of CD4 T cells-mediated immune responses to self-antigens such as HSP-60 and oxidized low density lipoprotein (OxLDL) [17], [18], [19]. In this study, we found that the proportion of Th1 and Th17 cells continued to rise concomitant with atherogenic processes in Apo E^−/−^ mice, however, we found that the numbers of circulating Treg continuously increased. Moreover, the immunosuppressive capacity of Treg from Apo E^−/−^ mice was comparable to the counterparts from wild-type littermates, both Treg could significantly suppress cell proliferation and cytokine production of effector T cells.

Combined the fact that Tregs were present in high numbers and fully functional with the phenomenon that effector T cells mediated-inflammation persisted in vivo, we supposed that some inflammatory-related factors caused resistance of effector T cells to suppression that contributed to the ongoing autoimmune response. It has been reported that IMC accumulation occurs in several autoimmune diseases, but there is still an ongoing debate that these cells play a pro-inflammatory or anti-inflammatory role in autoimmune diseases [20], [21]. We found that the frequency of IMC significant increased in the spleen of atherosclerotic Apo E^−/−^ mice. In vitro co-culture assay, we found that IMC did not directly change the proliferation level of CD4^+^CD25^−^ T cells, but responsiveness of CD4^+^CD25^−^ T cells to suppression by Treg was significantly impaired the presence of IMC isolated from atherosclerotic Apo E^−/−^ mice. Moreover, the vigorous proliferation of effector T cells was observed in atherosclerotic Apo E^−/−^ mice whereas depletion of IMC dramatically reduce the percentage of proliferating cells. To our knowledge, our findings first showed that IMC had the potential to induce unresponsiveness of pro-inflammatory T cells to suppression in atherosclerosis.

The important role of IL-6 in atherogenic processes has been extensively studied [22]. IL-6 acts as a pleiotropic cytokine directly affects multiple cell population, such as immune cells, smooth muscle cells, and endothelial cells. High level of serum IL-6 may result in activation of inflammatory cells, dysfunction of vascular endothelial cells, and abnormal proliferation and migration of vascular smooth muscle cells, thereby accelerating the development of atherosclerosis [23]. We found that IMC from atherosclerotic Apo E^−/−^ mice secreted significantly high level of IL-6 compared with the counterparts from tumor-bearing mice, and the serum level of IL-6 was significantly down-regulated by depletion of IMC in atherosclerotic Apo E^−/−^ mice. We further demonstrated that IL-6 was the main effective mediator of IMC to induce unresponsiveness of pro-inflammatory T cells to suppression because blockade of IL-6 could significantly abolish IMC-induced unresponsiveness of CD4^+^CD25^−^ T cells to suppression by Treg. Furthermore, the serum levels of INF-γ and IL-17A were significantly down-regulated by either depletion of IMC or blockade of Jak/Stat signaling pathway in atherosclerotic Apo E^−/−^ mice. Taken together, these findings might provide new insights into the immunopathogenesis of atherosclerosis and the exploration of potential targets for therapeutic intervention.
